# Metastatic Renal Cell Carcinoma and Unforeseen Adrenal Insufficiency: A Case Report and Literature Review

**DOI:** 10.7759/cureus.35265

**Published:** 2023-02-21

**Authors:** Matthew L Repp, Rodrigo A Alvarez, Dory E Arevalo-Salazar, Rajesh Kotagiri

**Affiliations:** 1 Medical School, University of Arizona College of Medicine, Tucson, USA; 2 Internal Medicine, University of Arizona College of Medicine, Tucson, USA; 3 Pathology, University of Arizona College of Medicine, Tucson, USA

**Keywords:** adrenal gland injury, metastatic kidney cancer, adrenal insufficiency, renal cell carcinoma, genitourinary oncology

## Abstract

Renal cell carcinoma (RCC) can metastasize to nearly every organ, yet rarely metastasizes to the adrenal glands despite their anatomical proximity. Adrenal metastases are typically incidental findings on medical imaging and are vastly clinically asymptomatic. The adrenal glands can maintain hormonal homeostasis if a tenth of total adrenal gland function is preserved. We present a patient with synchronous bilateral adrenal metastases from RCC with rapid and unexpected development of adrenal insufficiency (AI).

## Introduction

Renal cell carcinoma is the most common malignant neoplasm of the kidneys, accounting for approximately 85% of all renal cancers. At the time of diagnosis, nearly one-third of patients will have metastatic disease, most commonly involving the lung (45%), bones (30%), and lymph nodes (22%) [1]. Despite the proximity of the adrenal glands to the kidneys, RCC infrequently spreads to endocrine organs. Adrenal metastases are typically asymptomatic and found incidentally on imaging. These metastatic lesions can be categorized as synchronous or metachronous, as well as ipsilateral, contralateral, or bilateral. Although still rare, ipsilateral adrenal metastasis is relatively more common than contralateral spread, while bilateral adrenal metastasis is exceedingly rare. An autopsy study including 1,828 deceased patients with RCC observed isolated metastatic disease in the ipsilateral adrenal gland in 2.5% of individuals and contralateral adrenal gland metastasis in 0.7% [2]. Two large studies cumulatively evaluated metastatic sites of over 20,000 patients with RCC and reported metastases to the adrenal glands in only 9% of patients, without distinguishing laterality [1,3]. Based on a thorough review of the literature, adrenal insufficiency (AI) from metastatic renal cell carcinoma (mRCC) is markedly rare. Here we present a patient with synchronous bilateral adrenal metastases from mRCC with rapid and unexpected development of AI.

## Case presentation

A previously healthy 75-year-old man was hospitalized for severe bilateral lower back pain which was worse on the right side. History revealed a 20-pound unintentional weight loss over several months. He was still able to ambulate and denied any history of back trauma, neurologic symptoms, or bowel and bladder incontinence. He reports being a lifetime nonsmoker but did have significant occupational exposure to petroleum by-products throughout his aviation career. 

**Figure 1:**
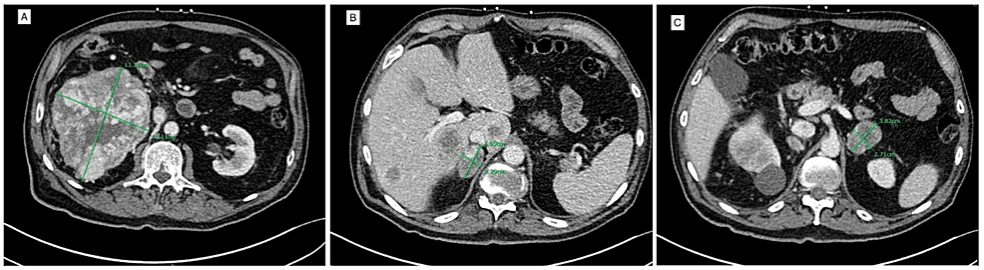
(A) Axial view of the abdominal CT scan demonstrating a heterogeneous hyperenhancing mass in the right kidney measuring 10.9 x 13.1 x 11.4 cm, diagnosed as renal cell carcinoma. (B) Heterogeneous enhancing mass in the right adrenal gland measuring 3.9 x 2.4 cm. (C) Heterogeneous enhancing mass in the left adrenal gland measuring 3.8 x 2.7 cm.

At hospitalization, the patient was afebrile and hypertensive to 175/80. Complete blood count and iron studies were significant for a hemoglobin of 13.3 g/dL (normal 13.5 to 17.0), low iron, low transferrin, low total iron-binding capacity, and elevated ferritin to 1,907 ng/mL (normal 25 to 506) consistent with anemia of chronic disease. Blood chemistry studies were significant for creatinine of 1.52 mg/dL (normal 0.6 to 1.5), glomerular filtration rate (GFR) of 47 mL/min/1.73 m2 (normal >60), blood urea nitrogen (BUN) of 32 mg/dL (normal 8 to 25), calcium of 11.0 mg/dL (normal 8.8 to 10.4), and alkaline phosphatase of 170 U/L (normal 40 to 140). Besides hypercalcemia, all other electrolytes were within normal limits. Computer tomography (CT) of the thorax, lumbar, chest, abdomen, and pelvis showed a large, heterogenous, hyperenchancing renal cell carcinoma along the inferior pole of the right kidney with bilateral adrenal metastases (Figure 1A-1C).

**Figure 2:**
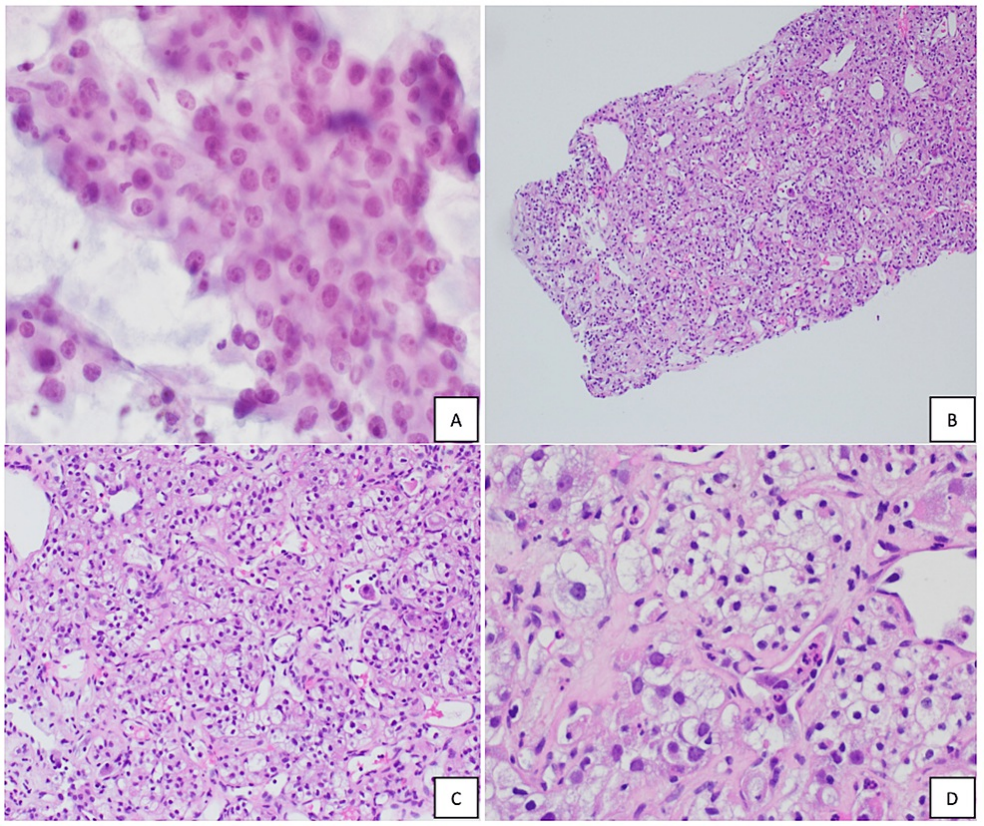
Pathological examination of the right renal mass showing clear cell renal cell carcinoma. (A) Photomicrograph showing a hypercellular specimen characterized by enlarged cells with prominent nucleoli, slight nuclear contour irregularities, granular cytoplasm, and some intricate transgressing blood vessels. (B) H&E section at 10x magnification showing low N:C ratio cells with clear, vacuolated cytoplasm and delicate arborizing vascular networks. (C) H&E at 20x magnification showing eccentric nuclei and clear, vacuolated cytoplasm. (D) H&E at 40x magnification showing malignant cells in a trabecular pattern with eccentric nuclei and clear, vacuolated cytoplasm.

There was also evidence of pulmonary, hepatic, pancreatic, and osseous metastases with pathologic fracturing at T11, T12, L4, and S1 vertebrae. An ultrasound needle-guided biopsy of the right renal mass was performed and deemed to be clear cell renal cell carcinoma (ccRCC) by pathology as can be seen in Figure 2A-2D.

The patient's pain was controlled with acetaminophen, topical lidocaine, methocarbamol, and hydromorphone, and he was discharged with a scheduled outpatient genitourinary oncology appointment the following week.

Two days later, the patient presented back to the emergency department with malaise, fever (39.3°C), tachycardia (115 bpm), and hypotension (88/70 mmHg) refractory to fluids. Complete blood count and metabolic panel were relatively unchanged from the previous admission. He was given norepinephrine for hemodynamic support and empirically started on cefepime, vancomycin, and metronidazole for suspected sepsis. Antibiotics were discontinued after a thorough sepsis workup was performed with negative findings. There were no significant lab findings on readmission. Serum cortisol levels the next morning (8 a.m.) were 4.4 $\mu$g/dL (normal 4.8 to 19.5) which prompted stress dosing with hydrocortisone 100 mg followed by maintenance treatment with 50 mg every six hours. The patient’s clinical status rapidly improved and his hemodynamic stability allowed weaning of norepinephrine. Endocrinology was consulted and recommended tapering hydrocortisone over the next three days and performing an adrenocorticotropic hormone (ACTH) stimulation test on the fourth day. Conclusively, the patient failed the ACTH (cosyntropin) stimulation test, as can be seen in Table 1, and was subsequently diagnosed with carcinoma-related AI. He was discharged on hydrocortisone 10 mg in the morning and 5 mg at night with close endocrinology and oncology follow-up.

**Table 1:** ACTH (cosyntropin) stimulation test resulting in cortisol levels <18 $\mu$ ​​​​​​​g/dL at 30- and 60-minutes post administration of 250 $\mu$ ​​​​​​​g of cosyntropin, demonstrating secondary adrenal insufficiency.

Test	Result	Reference Range
8:00 AM cortisol	4.1 $\mu$g/dL	4.8 - 9.5 $\mu$g/dL
Baseline ACTH	14 pg/mL	6 - 50 pg/mL
30-minute cortisol	8.8 $\mu$​​​​​​​​​​​​​​g/dL	>18 $\mu$​​​​​​​​​​​​​​g/dL
60-minute cortisol	10.6 $\mu$​​​​​​​​​​​​​​g/dL	>18 $\mu$​​​​​​​​​​​​​​g/dL

## Discussion

Adrenal glands demonstrate an immense ability to compensate, only requiring 10% of functioning glands to maintain hormonal homeostasis [4]. Most patients with adrenal metastases will never experience symptoms related to endocrinological dysfunction and are therefore not recommended to go on prophylactic hormone replacement. The most effective treatment of metastatic disease is the treatment of primary cancer with systemic therapy. We present a patient with ccRCC with known adrenal gland metastases who was discharged from the hospital in a stable condition and then rapidly and unexpectedly went into an adrenal crisis. The unforeseen progression into AI was found to be unparalleled in the literature.

Based on a thorough PubMed review of the literature, there were a total of 24 accessible case reports in English of bilateral adrenal metastasis from RCC, ours accounting for the 25th as can be seen in Table 2.

**Table 2:** Reported cases of bilateral adrenal gland metastases from renal cell carcinoma. B = bilateral, L = left, Met = metachronous, R = right, Syn = synchronous

Author, Year	Sex, Age (Years)	Primary Site	RCC subtype	Discovery Time	Symptoms	Adrenal Insufficiency
Zornoza and Bernardino (1980) [5]	-	-	Unspecified	-	-	-
Goffman et al. (1982) [6]	M, 48	R	Unspecified	Syn	Fever, orthostatic hypotension, dehydration	Yes
Luciani et al. (1985) [7]	M, 60	L	Unspecified	Met (5 years after left radical nephrectomy)	Abdominal pain/nausea	No
Selli et al. (1987) [8]	M, 61; M, 74; M, 63	B; R; L	Unspecified; Unspecified; Unspecified	Syn; Syn; Syn	Painless hematuria; Malaise, emesis; Fever, anorexia	No; No; No
Yu et al. (1992) [9]	M, 76	L	Clear cell carcinoma	Syn	Left flank pain	No
Schomer and Mohler (1995) [10]	-, 42	R	Clear cell carcinoma	Syn	Asymptomatic	No
Tsuboniwa et al. (1995) [11]	M, 72	L	Clear cell carcinoma	Syn	Complications from ureteral stenosis after prior gastrointestinal procedure	No
Tsukamoto et al. (1998) [12]	F, 58; M, 68	L; R	Clear cell carcinoma; Clear cell carcinoma	Syn; Syn	-; -	No; No
Koutalellis (2009) [13]	F, 58	R	Clear cell carcinoma	Syn	Right flank pain	No
Wu et al.(2010) [14]	M, 70	R	Chromophobe	Met (6 years after right-radical nephrectomy)	Left intrascrotal enlargement	No
Moslemi et al. (2010) [4]	M, 72	L	Clear cell carcinoma	Syn	Hematuria	No
Yoshino et al. (2012) [15]	M, 72	L	Unspecified	Met (4 years after left-radical nephrectomy)	Asymptomatic (found during surveillance check-up)	Subclinical to clinically significant adrenal insufficiency on Sunitinib
Kravvas et al. (2014) [16]	F, 58	L	Clear cell carcinoma	Syn	Frank hematuria Urethral meatus nodule	No
Ozturk (2015) [17]	M, 50	R	Clear cell carcinoma	Syn	Right flank pain	No
Ozturk (2015) [18]	M, 61	L	Clear cell carcinoma	Met (2 years after left-radical nephrectomy)	Asymptomatic (found during surveillance check-ups)	No
Costantino et al. (2016) [19]	M, 68	-	Unspecified	Syn	Fatigue, anorexia, postprandial nausea, and unintended weight loss	No
Nouralizadeh et al. (2017) [20]	F, 64	L	Clear cell carcinoma	Met (7 years after left radical nephrectomy)	Asymptomatic	No
Jimenez et al. (2018) [21]	F, 54	L	Clear cell carcinoma	Syn	Vaginal bleeding	No
Pandey et al. (2018) [22]	F, 60	B	Clear cell carcinoma	Syn	Hematuria	No
Ueda et al. (2019) [23]	F, 72	L	Clear cell carcinoma	Syn	Hematuria	No
Li et al. (2021) [24]	F, 64	L	Unspecified	Met (4 years after left-radical nephrectomy)	Asymptomatic (found during surveillance check-up)	No
Current report, 2022	M, 75	R	Clear cell carcinoma	Syn	Severe back pain and shock	Yes

There were approximately a dozen more case reports that were inaccessible due to language barriers. Approximately 58% of the RCC that spread to the bilateral adrenal glands were of the clear cell subtype. Only two cases were diagnosed with adrenal insufficiency [6,15]. The first case described a patient that developed a selective mineralocorticoid insufficiency, and the autopsy revealed a complete replacement of the right adrenal gland by RCC, with a near-complete replacement of the left adrenal gland [6]. The second case showed laboratory evidence of subclinical adrenal insufficiency and developed clinical AI during treatment with sunitinib [15]. To the best of our knowledge, we present the first case of a true adrenal crisis from mRCC to the bilateral adrenal glands.

Adrenal involvement is an ominous prognostic factor. AI in malignancy may be overlooked given the overlap of nonspecific clinical symptoms between the two conditions such as weight loss, anorexia, fatigue, nausea, and vomiting. There are currently no clear guidelines regarding prophylactic medical management or screening protocols for patients with adrenal metastases. AI screening on all patients with adrenal metastases with ACTH stimulation testing is theoretically possible, yet its utility is equivocal [25]. The ACTH stimulation test is the gold standard for diagnosing AI and is especially useful when there is overt evidence of AI in the context of malignancy, but it is unable to provide evidence-based recommendations for nuanced cases such as ours. The adrenal system becomes stressed as metastatic disease burdens the body, which can mask an impending adrenal crisis, further complicating diagnostic accuracy [25]. Several studies also describe patients with signs and symptoms of clinical AI with bilateral adrenal metastatic disease, yet do not meet diagnostic criteria based on biochemical or laboratory findings [26-27].

In hindsight, the patient’s extensive adrenal gland destruction was evident on imaging, making a future adrenal crisis not unlikely. The lack of guidelines for managing and predicting endocrinological emergencies in these patients is concerning given the lethality of an adrenal crisis. Future studies are needed to develop evidence-based approaches regarding screening and prophylactic pharmacological interventions for patients with bilateral adrenal metastases. The goal should be to create screening and surveillance guidelines for AI, as well as imaging criteria such as tumor size and characteristics that can help with risk stratification.

## Conclusions

We report to the best our knowledge, the first case of renal cell carcinoma metastasizing to the adrenal glands leading to adrenal insufficiency and an adrenal crisis. Currently, the standard of care for treating bilateral adrenal metastases is to treat the primary neoplasm with systemic therapy. In the setting of malignancy, adrenal crisis is unpredictable and life-threatening. The lack of research and clinical guidelines on medical management of bilateral adrenal metastases is concerning and prompts future action to be taken to improve patient care and outcomes.
